# Dopaminergic Signaling: Driver of Mobility Resilience and Moderator of Response to Pharmacotherapy

**DOI:** 10.1093/geroni/igaa057.2726

**Published:** 2020-12-16

**Authors:** Caterina Rosano, Andrea Rosso, Nicolaas Bohnen

## Abstract

Although effective mobility is the end result of the functional capacity of multiple systems, some adults appear resilient and maintain higher mobility later in life. Epidemiological studies suggest up to 20% of adults aged 65 and older maintain fast walking speed in the presence of multi-system impairments. What could be the reason for such mobility resilience? In this symposium, we focus on the brain dopaminergic signaling as a potential driver of mobility resilience. We examine the genetic val158met polymorphism of catechol-O-methyltransferase (COMT), an enzyme regulating dopaminergic signaling in the brain. The Met allele results in higher tonic dopamine levels; the Val allele results in lower tonic dopamine levels. The influence of COMT polymorphism on mobility is known for Parkinson’s disease (PD) and neurodegenerative conditions, but it not clear for asymptomatic adults aged 65 living in the community. We present data from the Cardiovascular Health Study, the Health Aging Body Composition Study, and several neuropharmachological studies with extensive information on PD ascertainment, medications, and health characteristics. We test the hypothesis that the Met allele of the COMT gene is associated with a) lower fall risk (Rosso), b) slower decline in gait reserve (Sprague), c) less gait slowing due to frailty (Mannon), d) more favorable response to pharmacotherapy in older adults without PD (Bohnen). Our results suggest a unique modulatory capacity of dopaminergic-related genes that may favor mobility resilience and predict better response to therapy. Our data provide clues for novel targeted interventions to delay physical disability in older adults.

